# Study of respiratory chain dysfunction in heart disease

**DOI:** 10.15171/jcvtr.2018.01

**Published:** 2018-03-17

**Authors:** Seyyed Hossein Hassanpour, Mohammad Amin Dehghani, Seyyedeh Zeinab Karami

**Affiliations:** ^1^Young Researchers and Elite Club, Yasooj Branch, Islamic Azad University, Yasooj, Iran; ^2^Department of Toxicology, School of Pharmacy, Ahvaz Jundishapour University of Medical Sciences, Ahvaz, Iran; ^3^Department of Biology, School of Basic Sciences, Yasouj University, Yasouj, Iran

**Keywords:** Beating Heart, Metabolic, Respiratory Chain, Mitochondrial, Cardiac Disorders

## Abstract

The relentlessly beating heart has the greatest oxygen consumption of any organ in the body at rest reflecting its huge metabolic turnover and energetic demands. The vast majority of its energy is produced and cycled in form of ATP which stems mainly from oxidative phosphorylation occurring at the respiratory chain in the mitochondria. A part from energy production, the respiratory chain is also the main source of reactive oxygen species and plays a pivotal role in the regulation of oxidative stress. Dysfunction of the respiratory chain is therefore found in most common heart conditions. The pathophysiology of mitochondrial respiratory chain dysfunction in hereditary cardiac mitochondrial disease, the aging heart, in LV hypertrophy and heart failure, and in ischaemia-reperfusion injury is reviewed. We introduce the practicing clinician to the complex physiology of the respiratory chain, highlight its impact on common cardiac disorders and review translational pharmacological and non-pharmacological treatment strategies.

## Introduction

Approximately 25% of a human myocardial cell is made up of mitochondria. Mitochondria are cellular factories converting substrates from diet into usable energy for many intracellular processes including mechanical contraction of myofilaments. The ultimate substrate used by most enzymes to convert chemically stored energy into conformational changes and finally mechanical motion is adenosine-triphosphate (ATP). The heart has a voracious requirement for energy – indeed the human heart cycles approximately 6kg of ATP per day.^1^ The majority of this ATP is generated in mitochondria at the respiratory chain by oxidative phosphorylation, and as a byproduct the respiratory chain generates reactive oxygen species (ROS). Under physiological conditions ROS plays an important role in intracellular signalling, but in pathological states increased ROS production can become detrimental to the cardiomyocyte. Associated with energy balance are other mitochondrial key roles, namely regulation of calcium homeostasis and apoptotic signalling. It is beyond the scope of this review to discuss in detail the latter two important processes. It is not surprising that mitochondrial diseases preferentially affect tissues with high energy turnover such as the heart. Impaired oxidative phosphorylation and defective electron transport chain (ETC) function are central to most cardiac conditions associated with mitochondrial dysfunction. Their malfunction has been implicated in hereditary mitochondrial cardiomyopathies, in the aging heart, cardiac hypertrophy, heart failure, and in ischaemia-reperfusion injury.

## Review method

In this study, we reviewed papers related to Respiratory Chain Dysfunction in Cardiac Disease. For this purpose, we searched keywords such as beating heart, metabolic, respiratory chain, mitochondrial, cardiac disorder in databases include web of science, PubMed and Scopus since from 1992 to 2017.

## Physiology of respiratory chain

**Figure 1:**
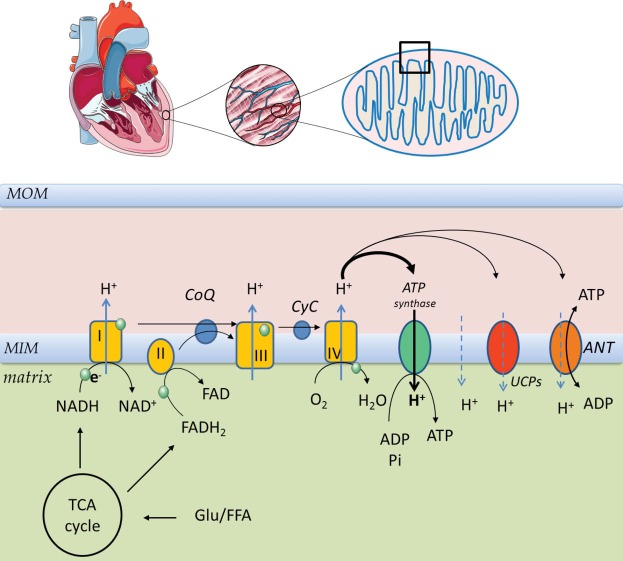
Mitochondrial respiratory chain and oxidative phosphorylation.
Under aerobic conditions substrates from fatty acid oxidation and glycolysis are metabolized in the TCA cycle which delivers high energy loaded electrons (in form of hydrogen via NADH and FADH_2_) to the electron transport chain (ETC) complexes located in the inner mitochondrial membrane. MIM mitochondrial internal membrane, CoQ coenzyme Q (ubiquinone), CyC cytochrome C, I/II/III/IV electron chain transport complexes I, II, III and IV; ANT adenine nucleotide translocase, UCPs uncoupling proteins.

**Figure 2:**
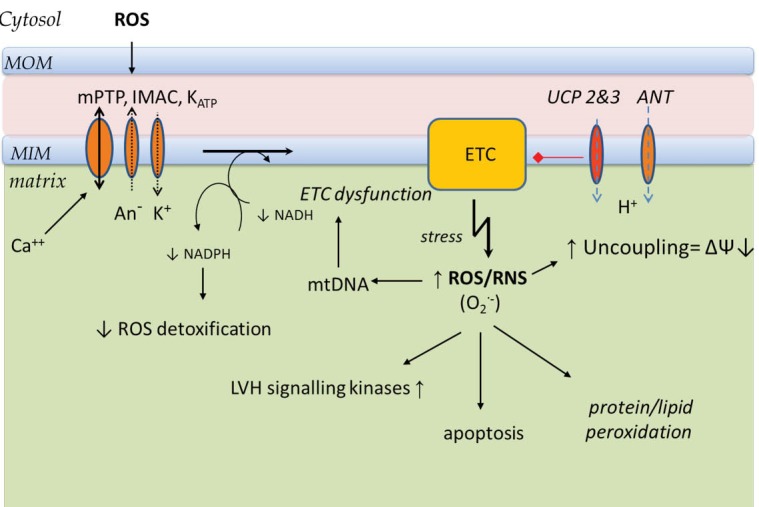
Pathophysiology of respiratory chain, reactive oxygen species production/signalling and oxidative phosphorylation, uncoupling, ‘ROS induces ROS’.
Inherited or senescence acquired mtDNA abnormalities, LVH or heart failure all can lead to dysfunction of ETC with increase of electron leak, mainly at complexes I and III and result in pathologic ROS production with detrimental consequences to the mitochondrion and the cell. ROS/RNS reactive oxygen/nitrogen species, O_2_- superoxide anion, mtDNA mitochondrial DNA, ETC electron chain transport system, ΔѰ mitochondrial internal membrane potential, IMAC inner membrane anion channel, mito-K_ATP_ mitochondrial ATP-dependent K+ channel, mtPTP mitochondrial permeability transition pore , UCP2 & UCP3 uncoupling proteins

Mitochondria generate ATP, by means of the ETC and the oxidative phosphorylation system (OXPHOS). The proteins involved in this process are located in the mitochondrial inner membrane (MIM) and collectively referred to as the respiratory chain (RC) (Figure 1). Acetyl CoA generated from glycolysis and from fatty acid beta oxidation (FAO) enters the tricarboxylic acid cycle (TCA). The TCA cycle, glycolysis and FAO all generate high energy electrons in the form of NADH (nicotinamide adenine dinucleotide). These electrons are then passed along the ETC in a series of redox reactions. The ETC comprises 5 protein complexes and two shuttles (Figure 1). NADH passes an electron to complex I (NADH dehydrogenase) and this in turn passes the electron through a shuttle coenzyme Q (Ubiquinone) to complex III (cytochrome b-c1). Another source of high energy electrons for complex III stems from FADH_2_ which is generated in the TCA cycle by succinate dehydrogenase which is both a TCA cycle component and a component of the ETC complex II. The final common pathway through complex III transfers electrons to another electron carrier, cytochrome C, which in turn passes its electrons to complex IV (cytochrome oxidase). Finally the energy depleted electron (in the form of hydrogen) is accepted by molecular oxygen, completely reducing it to form water. This series of redox reactions releases energy which drives the extrusion of protons outwards through complexes I, III, and IV to create an electrochemical gradient across the inner mitochondrial membrane. This gradient in turn drives the phosphorylation of ADP to ATP by ATP synthase (complex V), figure 1. This reaction is reversible and during severe ischaemia large amounts of ATP may be ‘wasted’ in maintaining the electrochemical gradient via the dephosphorylation of ATP. However OXPHOS is not fully efficient and even under physiological conditions some of the energy is dissipated as heat. This is due to proton leak from the inter mitochondrial membrane space into the matrix through uncoupling proteins (UCPs), adenine nucleotide translocase (ANT) or non-specific membrane proton slippage (Figure 1). This non-ATPase related loss of trans-membrane potential makes ATP production by OXPHOS less efficient (less ATP produced per oxygen molecule consumed). ANT catalyses the exchange of ADP and ATP between cytosol and mitochondria, but it also contributes to proton leak. UCP 2 and UCP3 are found in human cardiac muscle and their expression correlated positively with plasma free fatty acid concentrations.^2^ Their activities are increased by reactive oxygen species (ROS)^3^ (Figure 2). Physiological uncoupling of OXPHOS may decrease excessive ROS production and reduce oxidative damage (‘Uncoupling to survive’).^4^ Indeed rather than thermogenesis, this may be the major physiological role of uncoupling. Approximately 20%-30% of resting cellular energy expenditure dissipates as heat due to proton leak. In a negative feedback cycle ROS induced uncoupling leads in turn to suppression of ROS production by the ETC (Figure 2). The decreased trans-membrane potential (ΔѰ) regulates the ETC redox state, which in turn suppresses superoxide anion production by complexes Ⅰ and III.^5^ Superoxide production (O_2_-) is related to electron leak, which is closely interlinked with proton leak regulation. Electron leak can occur when electrons exit the ETC early before their final reduction to water to form superoxide instead. Under experimental conditions a high ΔѰ (or ΔpH) can reverse the electron transport at complex Ⅰ, reducing NAD^+^ to NADH and forming superoxide. An uncoupling induced decrease in the proton gradient reduces the reverse electron transport and the superoxide production at complex I.^6^ Mitochondria are thus both sources (complex Ⅰ and III) and targets of reactive oxygen and nitrogen species (ROS and RNS). Electron slippage at complexes I and III lead to incomplete reduction of molecular oxygen to form superoxide. At non-pathological levels ROS play important functions in cellular signalling. However when oxidative stress is increased the associated mtDNA damage may further enhance ROS production, resulting in a vicious cycle.^7^

## Hereditary cardiomyopathies

The RC system is made up of about 100 different proteins. Only 13 of these are encoded by mitochondrial DNA ([mtDNA] with a maternal pattern of inheritance),^8^ the remainder being encoded by nuclear DNA (nDNA), following a Mendelian inheritance pattern.^9^ All complexes of the ETC, except complex II which is encoded exclusively by mtDNA, have a double genetic origin (mtDNA and nDNA). Moreover it is hypothesised that several hundred nuclear genes are also needed for various functions of the RC.^10^ Increasingly it has also been recognised that mutations of mtDNA encoding for tRNA genes can affect protein synthesis with impaired RC function and lead to cardiomyopathy.^11^ The great variability of clinical presentation of inherited disorders related to mutations of mitochondrial genes is largely attributed to peculiar features of mitochondrial genetics, heteroplasmy and the threshold effect. A single mitochondrion can harbour both normal and mutant mtDNA- an effect known as heteroplasmy. A critical amount of mutant mtDNA is necessary to cause RC dysfunction and clinical symptoms (known as a ‘threshold effect’).^12^ Disease based epidemiology studies estimate the population prevalence of mtDNA disease at ~1:5000 while heteroplasmic mtDNA mutations are found in 1:200 of newborns (Elliot 2008). Mitochondrial disease can present at any age and affect almost any organ, but most commonly it involves the heart, brain, skeletal muscle, eye or endocrine system. Cardiologists need to consider the possibility of mtDNA disease if cardiac disease in the form of unexplained LV non-compaction, LVH, HCM, DCM or conduction defects are associated with maternal inheritance, either in isolation (e.g. HCM) or with other clinical features suggesting mitochondrial disease such as a combination of diabetes and deafness. Whereas syndromes like MELAS (myopathy, encephalopathy, lactic-acidosis, optic atrophy and stroke like syndrome) are well defined, many patients do not fit these syndromic categories.^13^ Barth syndrome, caused by impaired mitochondrial respiration was the first inherited disorder described as associated with left ventricular non-compaction, a rare congenital cardiomyopathy characterized by extensive endomyocardial trabeculation. However most LV non-compaction cardiomyopathies are caused by mutations of sarcomere genes overlapping with hypertrophic cardiomyopathy, rather than mitochondrial genes.^14^ Severe exercise limitation is typical of mitochondrial cardiomyopathies with associated skeletal myopathy and further investigation frequently reveals premature lactate acidosis during exercise. Massive proliferation of abnormal mitochondria with ragged red fibres on skeletal muscle biopsy and positive genetic testing contribute to the diagnosis.^12^ Rarely in predominantly cardiac involvement may endomyocardial biopsy become necessary.^13,15^

## Aging heart

In 1956 Harman suggested mitochondria as the main source of ROS and its causative role in age related changes.^16^ Short et al have confirmed that in human’s mtDNA abundance and ATP production declines with advancing age, whereas the level of oxidative mtDNA lesions increases.^17^ mtDNA is not protected by histones unlike nDNA and has less effective repair mechanisms.^18^ All of these factors contribute to a gradual increase in mtDNA mutation rates with age. This affects the expression and integrity of RC complexes which can lead to further ROS production perpetuating a vicious cycle of oxidative damage (Figure 2). A small age related decline in heart mitochondria numbers has been described in rats and humans, but this occurs without the loss of volume taken by mitochondria within cardiomyocytes. Aging cardiac mitochondria loose cristae and the RC function becomes impaired with lower average trans-membrane potential (ΔѰ), decreased ATP synthesis efficiency, and augmented ROS production with sensitization to mPTP opening which promotes apoptosis.^19^ These processes were previously linked to age related myocardial atrophy, stiffness and diastolic dysfunction.^19,20^ In animal models marked life span extension has been achieved by overexpression of enzymes which degrade ROS such as mitochondrial superoxide dismutases (MnSOD and Cu/ZnSOD)^21^ or catalase.^22^ In skeletal muscle over-expression of UCP3 leads to blunting of the age-induced increase in ROS,^23^ and animal models have confirmed the association of increased uncoupling with increased life span^24^ and improved mitochondrial biogenesis.^25^ However the vicious cycle proposed by the mitochondrial theory of aging, has been challenged by an experiment with mice expressing error-prone mtDNA polymerase.^26^ These mice accumulate substantial burdens of mtDNA mutations, associated with premature aging phenotypes and reduced life span. However their ROS production was normal and no increased sensitivity to oxidative stress-induced death was observed, despite severe RC dysfunction. The authors concluded that the mtDNA mutation accumulation with severe RC dysfunction per se is the primary inducer of premature aging independent of elevated ROS production. The ROS production may be merely a consequence, rather than driving force of the aging process.^26^

Dysfunctional mitochondria can trigger removal of damaged cells via apoptosis. However in non-proliferating tissues (such as heart) apoptosis of whole cells would be detrimental and therefore a more efficient system of mitochondrial quality control is necessary.^20^ The quality control happens by interplay of fusion, fission (splitting into two daughter mitochondria), autophagy (lysosomal break down of damaged proteins and organelles), and biogenesis of new mitochondria.^20^ Aging is associated with a decline in autophagy and accumulation of aberrant macromolecules in swollen giant mitochondria.^27^ Dysfunctional mitochondria with inhibited RC and depolarised membrane are unable to fuse with healthy mitochondria and later be targeted for removal by autophagy.^28^ Reduced autophagy in Atg5 (cardiac specific autophagy related 5 gene) deficient mice leads to age-related cardiomyopathy.^29^

## LV hypertrophy and heart failure

Changes in mitochondrial energetic profile are a hallmark of hypertrophied and failing hearts. Increased oxidative stress activates a variety of hypertrophy signalling kinases and transcription factors.^30,31^ Initially a pressure overload induced LV hypertrophy leads to a shift of fatty acid oxidation towards more efficient glucose oxidation. However it also leads to reduction of maximal OXPHOS capacity with decreased activities of RC complexes and increase of electron leak.^32^ At the failing heart stage the energy production decreases further and oxidative stress increases while facilitating cell dysfunction, and inducing apoptosis. ROS production also stimulates cardiac fibroblast proliferation, and expression and posttranslational activation of matrix metalloproteinases which play a pivotal role in extracellular remodelling. Oxidative stress can additionally activate apoptosis and contribute to maladaptive myocardial remodelling.^33^ The transition from compensated LV hypertrophy to failure is preceded by complex Ⅰ and II dysfunction followed by an increase of pro-apoptotic markers (Bax/Bcl-2 ratio).^34^ An increase in ROS production and other phenotypic similarities have been found in both the aging and the failing heart. Mitochondrial proteins as well as lipids may be targets of superoxide and its metabolites. This damage may lead to impaired mitochondrial respiration. Reduced maximal mitochondrial respiration was found in permeabilized cardiac muscle from dogs with ischaemia induced chronic heart failure^35^ and in patients undergoing cardiac transplantation.^36^ Some specific conditions including cumulative iron-mediated damage to mtDNA in hemochromatosis or myocardial inflammation in Chagas cardiomyopathy can alter structure, function and ETC activity leading to heart failure.^37-39^ Reduced activity of ETC subunits in patients with heart failure has been described previously, notably of complex Ⅰ,^40^ complex III^41^ and complex IV.^42^ These changes are found independently of the aetiology of the cardiomyopathy (ischaemic or idiopathic DCM). Impaired ETC activity can lead to increased mitochondrial ROS production.^43^ Additionally the elimination of ROS may be impaired as a marked decrease of MnSOD activity has been described in human failing heart.^44^ Recently it was suggested that cytosolic ROS may lead to amplification of mitochondrial ROS production (“ROS induces ROS”).^45^ The presence of mitochondrial nicotinamide adenine dinucleotide phosphate (NADPH) allows enzymatic detoxification of H_2_O_2_ and is in equilibrium with NADH produced by the TCA cycle. In the failing heart NADPH is more oxidized leading to increased mitochondrial H_2_O_2_ formation.^45^ Increased cytosolic ROS production can activate the mitochondrial permeability transition pore (mPTP), the inner membrane anion channel (IMAC) and the mitochondrial ATP-dependent K^+^ channel (mito-K_ATP_) found in the internal mitochondrial membrane. ANT is suspected as the core component of mPTP. Triggered by increased calcium, cyclophylin D induces such ANT conformational change that the mPTP complex becomes freely permeable to any molecule of <1.5kDa.^46^ This leads to dissipation of trans-membrane potential (ΔѰ) and subsequently to amplify electron flux through ETC (to maintain the ΔѰ) at the cost of increased NADH use. This may lead to increased NADPH oxidation and therefore impaired H_2_O_2_ detoxification. The opposite effect of ΔѰ dissipation on ROS accumulation by ‘physiologic uncoupling’ (ROS↓) versus cytosol ROS induced pathologic membrane depolarisation (ROS↑) needs clarification. However it is possible that while physiologic uncoupling regulates proton gradient by close modulation of electron flow and inhibition of ROS production at the ETC complexes, the excessive pathologic uncoupling by cytosolic ROS (by non-UCP channels and non-specific leak) lead to dramatically increased ETC flux necessary to maintain proton gradient (ΔѰ) and impaired ROS detoxification in the matrix. It is clear that ROS play an important role in the local pathogenesis of heart failure. This review focuses on the RC function and its major source of ROS production at the ETC of mitochondria; however it is important to highlight that there are other intracellular enzymatic sources of ROS such as NADPH oxidase, xanthine oxidase, and uncoupled nitric oxide synthases.^33^ Moreover circulating ROS metabolites could be also used clinically as a marker of heart failure severity and treatment efficiency. Biopyrrins, oxidative metabolites of bilirubin can be non-invasively measured in plasma or urine, and their levels correlated well to BNP levels and the severity of symptoms (NYHA class) in heart failure patients.^47^

## Ischaemia reperfusion injury – “To breathe or not to breathe?”

**Figure 3:**
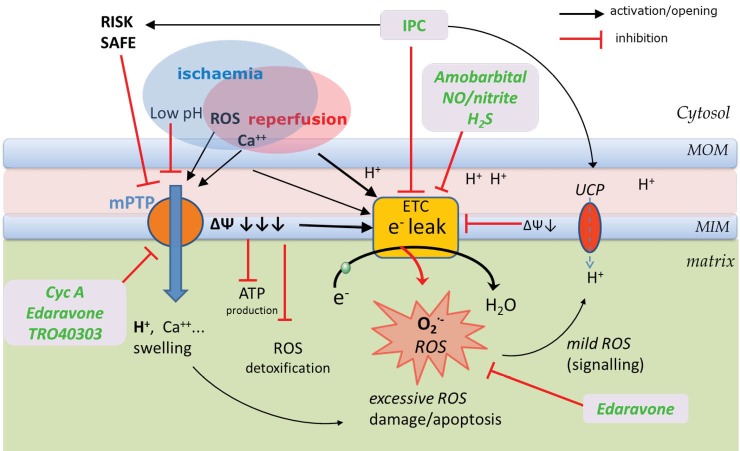
Ischaemia-reperfusion injury and therapeutic targets.
Ischaemia leads to impairment of mitochondrial OXPHOS related ATP production due to lack of O_2_ and decreased electrochemical gradient (ΔΨ) at the MIM. ROS reactive oxygen/nitrogen species, O2.- superoxide anion, ETC electron transport chain, mPTP mitochondrial permeability transition pore, UCP uncoupling proteins, MOM mitochondrial outer membrane, MIM mitochondrial inner membrane, RISK Reperfusion Injury Salvage Kinase pathway, SAFE Survivor Activating Factor Enhancement pathway, Cyc A cyclosporine A, NO nitric oxide, IPC ischaemic preconditioning.

Final infarct size is due to injury conferred during ischaemia and also the injury incurred as a result of ischaemia reperfusion injury (IRI). The damage occurring on reperfusion is largely determined by a massive burst of ROS production originating from ischaemically damaged mitochondria. During ischaemia intracellular ATP levels and pH drop due to impaired OXPHOS and a switch to anaerobic glycolysis with lactic acid production. The intracellular proton accumulation activates the Na/Hantiporter and sodium enters the intracellular space. The ATP dependent Na/K antiporter is now unable to remove the intracellular Na and excess Na leads to a reversal of the Na/Ca antiporter with a resultant increase of intracellular calcium and mitochondrial swelling. The Influx of calcium into the mitochondria and an increase in ROS production both favour the opening of the mitochondrial permeability transition pore (mPTP), but the associated low pH prevents its opening (Figure 3). ROS production during ischaemia is promoted by the accumulation of electrons within the ETC as hypoxia halts, or even reverses the electron flow. ETC complexes are in a reduced state which promotes acceptance of the electrons by the remaining oxygen to form superoxide.^48^ Following reperfusion pH recovers quickly towards normal and these results in opening of the mPTP within a few minutes of reperfusion.^49,50^ During early reperfusion and reoxygenation the ischemia damaged ETC in the presence of an abrupt increase in flow of accumulated electrons and an associated increase in electron leak (mainly at complex I and III) is responsible for a burst in superoxide production (Figure 3).^51-53^ Opening of the non-specific mPTP results in sudden dissipation of the electrochemical gradient across the inner mitochondrial membrane causing hydrolysis rather than synthesis of ATP and perpetuation of further ROS production which leads to irreversible oxidization of proteins, DNA, and lipids,^54^ release of cytochrome c and activation of apoptotic pathways.^49,55^ Reperfusion also results in local and systemic inflammatory reactions involving activation of neutrophils and platelets.^56^ The inhibition of mPTP opening has become a common final target for cytoprotective strategies in ischaemia-reperfusion injury.^46,57,58^ Cyclosporine A is a direct mPTP opening inhibitor and has been shown to decrease the infarct size following reperfusion in a pilot study of 58 patients presenting with acute STEMI (Figure 3).^46,59^ Another promising agent which is scavenging excess ROS and appears to inhibit mPTP opening is edaravone (MCI-186) (Figure 3).^60^ This antioxidant, approved for treatment of acute ischaemic stroke in Japan and China,^61^ was evaluated in a clinical trial in which it was administered 10 minutes before reperfusion in acute myocardial infarction and decreased size and preserved cardiac function (n = 80).^62^ TRO40303 is another novel cytoprotective agent acting via inhibition of mPTP opening^63^ which is currently being investigated for clinical use in acute myocardial infarction.^64^ Other experimental strategies comprise induction of upstream endogenous protective mechanisms by ischaemic conditioning or pharmacological targeting of upstream conditioning cascade of cytosol located pro-survival enzymes which inhibit mPTP opening such as RISK (Reperfusion Injury Salvage Kinase),^65^ SAFE (Survivor Activating Factor Enhancement) pathways^66^ by adenosine, opioids, ANP, PDE5 inhibitors and others and are reviewed in detail elsewhere.^57,67,68^ Activation of AMPK (adenosine mono-phosphate activated protein kinase) which beneficially modulates substrate transport and substrate oxidation in the reperfusion phase is another recognized pathway to prevent IRI.^69^ A novel strategy is modulation of the ETC and related mitochondrial ROS production to confer cytoprotection against myocardial injury on reperfusion and is discussed below. Ischaemic conditioning is a strategy to limit myocardial infarction size by induction of ischaemia either locally (by intermittent occlusion of the affected coronary vessel) or remotely in a distant organ (typically a limb) inducing myocardium cytoprotection. Ischaemic conditioning can be applied at different time points: before begin of ischaemia (PRE- conditioning, IPC), during ongoing myocardial ischaemia (PER-conditioning, IPerC) or at onset of reperfusion (POST-conditioning, IPostC). The ischaemia or pharmacological IPerC and IPostC strategies are clinically relevant in the setting of acute myocardial infarction^70,71^; whereas IPC has been successfully used in elective cardiac surgery or elective PCI setting where it reduced infarct size and improved post-ischaemic function.^72-75^ One of the cytoprotective mechanisms of IPC is an induction of a slight degree of MIM depolarization which protects against ROS induced damage.^76^ This mild proton leak induced by acute IPC is mediated mainly by UCPs (UCP 2 and 3).^77^ Late IPC leads to an increase in UCP2 expression. On its own the augmented uncoupling should impair energetic efficiency, however IPC also increases expression of complex IV and ATP synthase supporting ATP production with a favourable energetic profile during repeated hypoxia.^78^ Inhibition of complex I (a major ROS source) by acute IPC mediated by reversible s-nitration is another mechanism protecting from damage on reperfusion (Figure 3).^79^ There has been a surge in interest in the potential use of nitrite (NO_2_-) in the treatment of IRI. Under hypoxic conditions nitrite can be reduced to nitric oxide (NO), and is thought to act as the largest storage pool for the metabolically active NO.^80,81^ Cardioprotection is conferred during ischaemia by NO donors^82^ inhibiting both complex I (decreasing ROS production) and complex IV (Figure 3).^83,84^ Under hypoxic conditions, nitrite is reduced to NO and similarly to ischaemic preconditioning confers cytoprotection via blockade and S-nitrosation of complex I in mouse heart during ischaemia and reperfusion.^79,82,85,86^ We and others are currently undertaking phase II trials investigating the cytoprotective effects of iv nitrite in early reperfusion phase in patients with ST-elevation myocardial infarction (Table 1).^87,88^ Amobarbital, a complex l inhibitor, preserves mitochondrial respiration and decreases myocardial injury both during ischaemia,^89^ and during early reperfusion. It attenuates ROS generation with consequent decrease in infarct size (Figure 3).^90,91^ Unfortunately despite strong animal experimental evidence of its cytoprotective properties, exerting its action even in aged hearts lacking upstream signalling pathways of post-conditioning,^92^ this barbiturate narcotic historically used as a “truth serum” has not found its way to human IRI studies yet. Multiple other therapies are currently developed for the treatment of ischaemic heart disease and IRI; however they often do not target the RC directly and are reviewed elsewhere.^93^

**Table 1:** Translational strategies targeting the respiratory chain in cardiac disease

**Clinical Usage**	**Intervention**	**Target**	**Clinical Trials**	**Ref**
Ischaemia-reperfusion injury	Ischaemic conditioning	Complex I, IV; increased uncoupling, RISK, SAFE	AMI, remote preconditioning n=142, myocardial salvage index by perfusion imaging, *P*=0.03	^71^
			Elective CABG, remote preconditioning, n=57, Trop-T_AUC_, *P*=0.005	^73^
			Valve replacement surgery, remote preconditioning, n=81, Trop I_AUC_, *P*=0.05	^72^
	Nitrite	Complex I and IV	Currently undergoing- NIAMI,^.^multi-centre RCT (iv nitrite) NITRITE-AMI, single centre RCT (intracoronary nitrite)	NCT01388504 NCT01584453
	Melatonin	Stabilizes MIM preserving complex I and III function	currently undergoing-single centre MARIA (iv in AMI) two centre (intracoronary in AMI)	NCT00640094 NCT01172171
	Cyclosporine A	mPTP	AMI, n=58, CK_AUC_*P*=0.04, trop I_AUC_*P*=0.15, MRI *P*=0.04	^59^
	TRO40303	Mitochondrial translocator protein (TSPO), delays mPTP opening	Multi-centre RCT	NCT01374321
	Edaravone (MCI-186)	ROS scavenger	AMI, n=80, CK_AUC_*P*=0.04, CK-MB_AUC_*P*=0.02	^62^
	Other interventions showing benefit in animal models: Amobarbital,Hydrogen sulfide, Caloric restriction,Resveratrol	Complex I Complex IV Complex I and III Complex I and III		^90,91,134^ ^135^ ^128^
Heart failure	Coenzyme Q 10	Corrects coenzyme Q deficit	Coenzyme Q10 + Selenium, 5 year follow up, n=443, CV mortality *P*=0.015, NT-proBNP 0.014, EF 0.03	^103^
			Symbio multi-center RCT	ISRCTN945062
	Other interventions showing benefit in animal models: Trimetazidine^105^ SS-31^109^	Complex I and II Mitochondria selective antioxidant		^117^ ^122^
LVH	Animal model: Low intensity aerobic exercise	Decreased sensitivity to Ca^++^ induced mPTP opening		^129^
Aging	Animal models: Caloric restriction, Melatonin	Complex I electron leak		^126^
		Preservation of complex I, III , IV activity		^109^
Hereditary mitochondrial cardiomyopathies	Pre-natal genetic diagnostics & Gene therapy Low intensity exercise	Respiratory chain defects	(Benefit documented in skeletal muscle)	^136^

## Other potential therapeutic interventions targeting the respiratory chain

Ischaemia–reperfusion injury is a classic example where modulation of RC function has been extensively investigated in an experimental setting and currently significant efforts are undertaken to translate these results into human applications. However as described in the previous sections, RC dysfunction occurs in almost every pathology involving the working heart. Therefore it is not surprising that attempts to modify the ETC in order to improve myocardial energetics and limit oxidative stress damage are increasingly being investigated in other conditions as well (Table 1).

### Nitrite and nitrate

Inorganic nitrite (NO_2_-) or nitrate (NO_3_-) induced modulations of RC could be potentially beneficial for treatment of peripheral arterial disease,^94^ angina or heart failure.^80^ There is limited evidence that it may induce energetically favourable state and improve improved metabolic efficiency. In liver mitochondria NO induces depression of the maximal OXPHOS dependent ATP synthesis and this has been attributed mainly to inhibition of complex I and complex IV. The NO induced kinetic constraint on complex IV is however more pronounced than the constraint on ATP synthesis leading to improved oxidative phosphorylation efficiency (amount of ATP produced per oxygen molecules consumed).^95^ Though this inhibition may overall result in restricted maximal ATP synthesis capacity which could be detrimental in the highest metabolic demand, it may well be beneficial in situations when hypoxia limits oxygen supply and promote cardiac hibernation (Figure 3). There is emerging evidence that nitrate/nitrite improves metabolic efficiency of skeletal muscle of healthy volunteers by decreasing oxygen consumption at exercise.^96,97^ It was proposed that nitrite may improve coupling of OXPHOX to ATP synthesis, and therefore the efficiency of ATP synthesis.^96-98^ This increase in metabolic efficiency may be partly responsible for beneficial effects seen in peripheral artery disease patients with prolonged walking distances after beetroot juice nitrate supplementation.^94^ If similar action was present in cardiac muscle this could open up strategies to develop effective treatment for chronic heart failure patients and angina. However some of the benefits are likely due to previously demonstrated nitrite induced vasodilation of hypoxic tissue^99^ and better local perfusion due to nitrite induced angiogenesis.^100,101^

### Coenzyme Q

Coenzyme Q10 is an important antioxidant and a part of the RC. Low Coenzyme Q10 levels have been documented in chronic heart failure.^102^ Its supplementation in smaller studies showed improvement in LVEF and cardiac output in HF patients. Long-term supplementation of a combination of selenium and coenzyme Q10 in an elderly Swedish population resulted in significant decrease of cardiovascular mortality. The effect was also evident on multivariate analysis when adjusted for risk factors such as heart failure class or ejection fraction.^103^ Recently the preliminary results of a multi-centre randomized control trial Q-Symbio were presented. 420 patients with severe heart failure (NYHA III-IV) were randomized to receive either CoQ10 or placebo. CoQ10 decreased the risk of MACE (= hospitalization, CV death, mechanical circulatory support or cardiac transplantation) from 14% to 25% and halved the risk of dying from all causes compared to placebo.^104,105^ The Q-Symbio data have to be regarded with caution as the full data are still to be published, however if it stands the post-publication peer-review then this could be a breakthrough for medication which act by augmentation of the energy production, rather than just inhibiting less effective pathways or preventing negative impact of pathologic remodelling in heart failure. Beer (even alcohol-free) inhibits enzymatic activity of complexes I and IV and decreases the oxidation of Coenzyme Q9 and Q10 in adriamycin treated rats leading to decreased damage of mitochondrial components and preventing mitochondrial dysfunction.^106^

### Melatonin

Melatonin is found in high concentration in mitochondria where it stabilizes the MIM and improves the activity of the ETC. It protects against ROS induced cardiolipin peroxidation which would otherwise promote cytochrome c detachment and mPTP opening.^107^ Melatonin protects myocardium from ischaemic reperfusion injury, lowering lipid peroxidation, preserving mitochondrial respiration, and preventing loss of function of complex I, and III and improves post-ischaemic haemodynamic function in isolated heart.^107^ The ongoing phase II trial MARIA is investigating if melatonin confers cardioprotection in patients presenting with myocardial infarction undergoing primary angioplasty.^108^ There may be also a role in protection against the consequences of ageing as chronic melatonin administration reduces oxidative damage and mitochondrial function in hearts from senescence-accelerated mice.^109^

### Trimetazidine

Trimetazidine (TZD), an anti-anginal drug has been shown to improve myocardial function in both patients with ischaemic heart disease^110^ or with idiopathic DCM^111^ while preserving an advantageous energetic profile.^112,113^ A favourable metabolic modulation^114^ by a switch from fatty acid oxidation to glucose oxidation via inhibition of long-chain 3-ketoacyl CoA thiolase activity^115^ may play only a part in the observed beneficial effects. Further evidence however suggests that modulation of the ETC may be pivotal in the cytoprotection conferred by TZD. It protects cardiomyocytes in animal models of IRI^116^ or HF^117^ by inhibition of Ca_2_^++^ induced mPTP opening. In myocytes from failing hearts an enhanced electron leak at complex II was suppressed by TZD and hence the ROS generation was attenuated; the restoration of the redox balance by TZD was accompanied by an improvement of impaired activity of complex I.^117^

### Antioxidants

Multiple antioxidant agents have been investigated for their potential to reduce cardiovascular events, via oxidative stress reduction. These include vitamin E^118,119^ or omega 3.^120^ Unfortunately while many of these treatments show beneficial effects under experimental conditions outcomes in human trials have been mixed and do not seem to translate into reduced mortality.^121^ One of the reasons for the lack of the antioxidant effects may be the recognized concept of compartmentalized signalling. Mitochondrial ROS signalling may be dependent on localised proximity to target molecules, which may not reflect changes in their global concentration or effects on different isoforms of target proteins (with opposite effects) found in different compartments.^42^ This is supported by findings showing amelioration of experimental angiotensin II induced cardiomyopathy by targeted mitochondrial ROS scavenging with SS-31 (a ROS scavenging peptide which accumulates > 1000 fold in mitochondria), but no effect of non-targeted ROS scavenger N-acetyl-cysteine (NAC) in the same experiment.^122^

### Caloric restriction, resveratrol and exercise

Caloric restriction (CR) is unique in that it has been shown to increase maximum life span in mammals,^123,124^ possibly via the induction of autophagic pathways and mitochondrial biogenesis,^125^ and reduction of complex I related ROS production.^126^ It can ameliorate aging-associated changes in human cardiac diastolic function.^127^ CR preserves post-ischaemic mitochondrial respiration and attenuates post-ischaemic mitochondrial H_2_O_2_ production.^128^ Treatment with resveratrol (natural polyphenol) mimicked the effect of CR attenuating ROS production in ischaemia and reoxygenation. Both CR and resveratrol appear to protect from oxidative stress by deacetylation of specific ETC proteins.^128^ Low intensity exercise is known to attenuate pathological LV remodelling in human heart failure. In a swine model of pressure overload low level aerobic exercise prevented LV hypertrophy and systolic function. These beneficial changes were accompanied by attenuation of mitochondrial dysfunction.^129^

## Future directions

A wealth of evidence is currently available to confirm the major role of mitochondrial respiratory dysfunction in metabolic disorders of the heart. An exciting novel approach to identify new cardioprotective agents is the use of high-throughput tests measuring cellular respiration following various stressors by screening blindly thousands of small molecules from commercially available chemical compound libraries.^130,131^ Identified candidates are then subjected to more rigorous bench testing. This approach can perpetuate finding of new agents targeting mitochondrial function. Unfortunately the reality is that the complex physiology of mitochondrial metabolism and artificial experimental methodology are among the main reasons why many previously hailed therapeutic strategies failed later in human experiments. Many experiments are performed on isolated mitochondria which although easier to obtain and work with, they lack the cellular context.^132^ Experiments assessing mitochondrial function in the context of whole permeabilized fibres are more challenging and also its physiology still leaves scope for error due to lack of organismal context.^132^ In respect of mitochondrial function and identification of the individual targets, proteomics and metabolomics approaches may prove crucial in the near future. The picture becomes even more complex when various disease models are used. One attempt to overcome the variation in experimental ischaemia-reperfusion models is CAESAR (Consortium for Preclinical Assessment of Cardioprotective Therapies).^133^ Its mission is to introduce the same systematic randomization, standardized protocols and statistical rigor to preclinical studies and bridge these to clinical trials. Similar structured approaches should be attempted for studies into other conditions such as heart failure models or the aging heart. Despite early days, and multiple previous failures to translate promising in-vitro data into clinical setting we are currently witnessing the first few therapies succeeding in their translation.

## Conclusion

Cardiac function is dependent on mitochondrial aerobic energy delivery by oxidative phosphorylation. However the RC complex is important not only in aerobic energy delivery, but also in regulation of oxidative stress and cell signalling. There is growing body of evidence suggesting pivotal role of RC dysfunction in pathogenesis of common cardiac conditions such as heart failure or IRI. Understanding the molecular biology of these conditions is the premise for successful development of therapeutic and preventative targets. Potential treatment strategies are currently being translated from the bench to the bedside.

## Competing interests

The authors declare that there is no conflict of interest regarding this study.

## Funding

This study was supported by the authors named in this article.

## Ethical approval

This research does not contain any studies with human participants or animals and was performed by the authors alone
